# Atomic Resolution Defocused Electron Ptychography at Low Dose with a Fast, Direct Electron Detector

**DOI:** 10.1038/s41598-019-40413-z

**Published:** 2019-03-08

**Authors:** Jiamei Song, Christopher S. Allen, Si Gao, Chen Huang, Hidetaka Sawada, Xiaoqing Pan, Jamie Warner, Peng Wang, Angus I. Kirkland

**Affiliations:** 1 College of Engineering and Applied Sciences, Nanjing University, Nanjing, 210093 People’s Republic of China; 2 Department of Materials, University of Oxford, Parks Road, Oxford, OX1 3PH UK; 3 Electron Physical Sciences Imaging Centre, Diamond Lightsource Ltd., Didcot, OX11 0DE UK; 4 JEOL Ltd, 1-2 Mushashino, 3-Chome, Akishima, Tokyo, 196 Japan; 5 Department of Materials Science and Engineering, and Department of Physics and Astronomy, University of California, Irvine, CA 92697 USA

## Abstract

Electron ptychography has recently attracted considerable interest for high resolution phase-sensitive imaging. However, to date studies have been mainly limited to radiation resistant samples as the electron dose required to record a ptychographic dataset is too high for use with beam-sensitive materials. Here we report defocused electron ptychography using a fast, direct-counting detector to reconstruct the transmission function, which is in turn related to the electrostatic potential of a two-dimensional material at atomic resolution under various low dose conditions.

## Introduction

In scanning transmission electron microscopy (STEM), a number of significant instrumental advances have been made including aberration correctors^1–4^, monochromators^5–7^ and high-coherence electron guns^8^, which have enabled 50 pm resolution imaging at 300 kV for non-radiation-sensitive materials^9^ in several geometries. However, due to strong interaction of electrons with the sample, various mechanisms including radiolysis, displacement damage, sputtering and hydrocarbon contamination^10,11^ frequently occur causing damage of the specimen during high-resolution observation. For beam-sensitive materials, such as organic and biological materials, zeolites and ceramics, radiation damage^10,12–15^ has often been a limiting factor for atomic-resolution STEM imaging. Several methods^16–19^ have been developed to reduce the electron dose in the STEM geometry, however, a large number of probe positions are still required for atomic-resolution imaging over useful fields of view.

Ptychography^20–22^ has been widely implemented in both light and X-ray optics. Unlike traditional single-shot diffraction imaging^8,23–25^, ptychography does not require prior information about the probe function and is not restricted by limited fields of view and non-unique solutions^23^. In electron optics, it has also led to widespread interest owing to its potential for super-resolution^26,27^, high phase sensitivity^28^, three-dimensional imaging^29^, and phase retrieval under low-dose conditions^30^. Using a highly convergent beam geometry, Nellist *et al*. have reconstructed phase information from silicon^27^, bilayer graphene^28^, and a carbon nanotube^31^ at atomic resolution by evaluating the overlap between the first- and zero-order scattered discs and Müller *et al*. have recently achieved 0.4 Å Abbe limited resolution in data recorded from MoS_2_ using an electron microscope pixel array detector (EMPAD)^32^. Focused probes are compatible with the conditions required for incoherent imaging and hence this geometry can be used to simultaneously record ADF images. However, an atomically resolved ptychographic reconstruction of a useful field of view requires a data set of diffraction patterns (DPs) acquired from a very large number of probe positions and due to limited detector speeds (>100 μs), the total sample exposure time is still much longer than that used to record standard STEM images. This constrains the use of this method for radiation resistant materials and often degrades the ultimate resolution due to sample drift and/or restricts the useful field of view. An alternative technique is to scan a defocused probe over the sample with substantially overlapped probe positions. A relatively small number of probe positions are then required to successfully reconstruct the phase with high spatial resolution^33,34^ over a wide field of view. Based upon simulated datasets, Müller *et al*.^32^ have theoretically compared various ptychographic modes at electron doses as low as 250 e^−^/Å^2^ and have shown that the defocused probe mode is, under certain circumstances better than the focused probe geometry at low dose. Combining a non-convex Bayesian optimization with defocused ptychography, Pelz *et al*.^35^ have theoretically demonstrated that biological macromolecules can be reconstructed from simulated data with a resolution of 5.4 Å at a dose of 20 e^−^/Å^2^. Both of these reports, using simulated data suggest that ptychography in the defocused mode is promising for imaging radiation sensitive samples with electrons at high resolution.

Experimentally, using defocused probe ptychography, atomic resolution reconstructions have only been demonstrated at high doses of 10^5^−10^6^ e^−^/Å^2^. Putkunz *et al*.^36^ reported ptychographic reconstruction of a boron nitride helical cone at 1 Å resolution with a dose of 1.76 × 10^6^ e^−^/Å^2^ and Putkunz *et al*.^36^ and D’Alfonso *et al*.^37^ have improved this resolution in the recovered exit wavefunction to 0.8 Å using CeO_2_ nanocrystal test samples. They have also examined electron ptychographic datasets recorded under various electron doses obtaining atomic resolution for <110> oriented CeO_2_ nanocrystals at a dose of 1.54 × 10^6^ e^−^/Å^2^ ^30^. Using a dose of 0.94 × 10^6^ e^−^/Å^2^, Wang *et al*.^34^ have reported the reconstructed phase of LaB_6_ resolving both light B atoms, together with heavy La atoms. Significantly, in all of these reports, an electron dose of the order of 10^6^ e^−^/Å^2^ have been used to ensure a sufficiently high signal-to-noise ratio (SNRs) for high resolution, which has limited the experimental demonstration of electron-ptychography applied to radiation-resistant samples.

A major advance in ptychographic data acquisition has come from the development of direct-detection cameras that offer electron counting and fast acquisition times^38,39^. These have significantly increased the detective quantum efficiency (DQE) and have thus improved the SNR in the recorded far field DPs, enabling the recording of weaker signals at higher scattering angles in each DP within a ptychographic dataset. This potentially enables higher resolution in ptychographic reconstructions, even under the constraints of the low electron dose required for beam-sensitive materials.

In the work reported here, a monolayer of MoS_2_ was used as a model system to study the effect of electron dose on the phase and resolution of reconstructions using defocused ptychography. Reconstructions at high resolution were experimentally demonstrated at a dose of 403 e^−^/Å^2^, indicating the viability for future applications in phase reconstruction of biological materials^35^ at high resolution without damage. However, we note that this dose is still an order of magnitude higher than that routinely used in structural biology albeit at the higher target resolution required to resolve individual Mo and S columns. This suggests that there is potential for a further reduction in the dose for lower resolution reconstructions.

## Experimental

**Figure 1:**
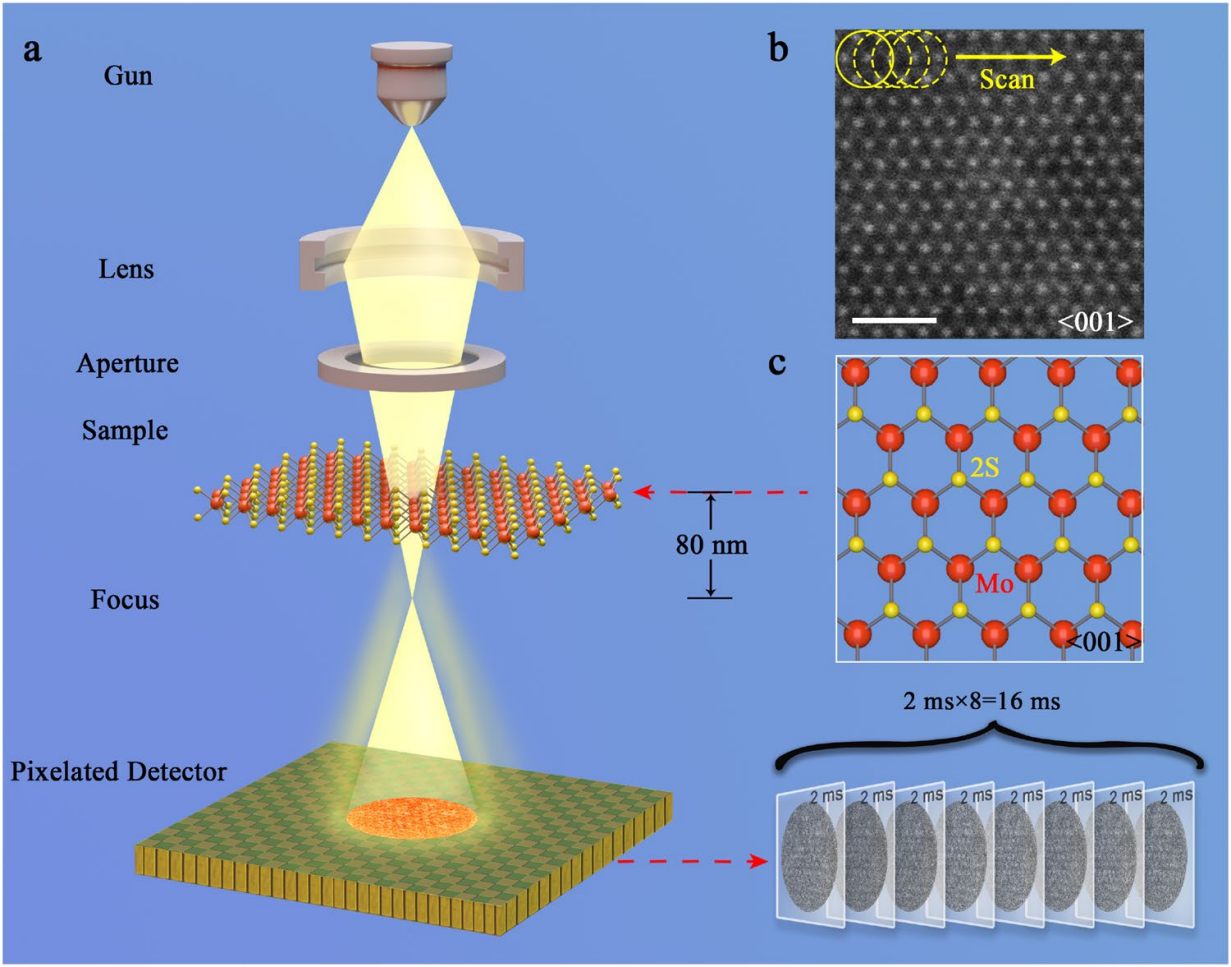
Illustration of the experimental setup. (**a**) Schematic of the experimental optical configuration used for ptychographic reconstruction. The sample was 80 nm above the beam focus. (**b**) HAADF image of a MoS_2_ monolayer oriented along <001> with the region where the reconstructed phases were restored using the ePIE algorithm indicated. The red circles represent the probe, and the arrow indicates the direction of the probe movement. Eight diffraction patterns were generated at each position of the probe scan. The acquisition time for each diffraction pattern was 2 ms. (**c**) Projected atomic models of MoS_2_ along <001>. The yellow balls represent sulfur atoms, and the red balls molybdenum atoms.

**Figure 2:**
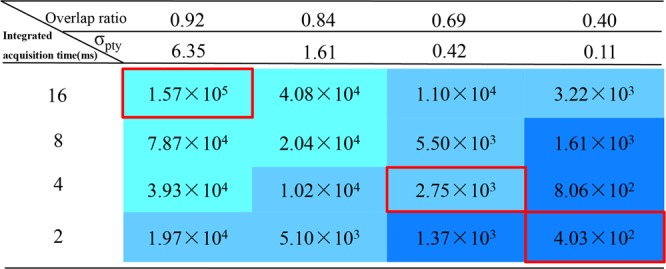
Variation in electron dose (e^−^/Å^2^) for different combinations of integrated acquisition times and overlap ratios. The array is divided into trichromatic regions based on the resolution achieved in each individual reconstruction. The light blue, blue and dark blue regions represent high-resolution (HR, 1.03 Å), mid-resolution (MR, 1.36 Å) and low-resolution (LR, 1.58 Å), respectively.

Experimental defocused electron ptychography was performed using a JEOL ARM300F instrument^40^ operated at 80 kV with a cold field emission source. Figure 1a shows a schematic diagram of the optical configuration. A probe-forming convergence semi-angle (α) of 24 mrad was used and the sample was placed ca. 80 nm above the focal point, such that the probe diameter at the sample plane was approximately 3.9 nm. A MoS_2_ monolayer oriented along <001> was illuminated by the probe in a 40 × 40 position raster scan with a 0.23 nm step size, as shown in Fig. 1b. This provided a maximum overlap ratio of 0.92 between adjacent probe positions. At each probe position, eight DPs, each with a 2 ms exposure time, were sequentially recorded on a Medipix3^38^ direct electron detector giving a total of 12800 DPs that could be used in an ePIE reconstruction^41^. A 17pA probe current was used corresponding to a dose of 1.78 × 10^2^ e^−^/Å^2^ per diffraction pattern. Since multiple DPs were recorded at each probe position, datasets could be synthesized post-experimentally by integrating different numbers of DPs at each probe position to give different doses^42^. Summations of eight, four, two, and one DPs provided four datasets with exposure times per DP equivalent to 16 ms, 8 ms, 4, ms, and 2 ms, respectively, as shown in Fig. 2. The advantage of this synthetic method is that it eliminates possible effects arising from external factors such as changes in beam current and sample drift. In conjunction with the variation of the integrated exposure time, the overlap ratio was also adjusted by selecting step sizes of 0.23 nm, 0.46 nm, 0.92 nm, and 1.84 nm for arrays of 40 × 40, 20 × 20, 10 × 10, and 5 × 5 DPs respectively, corresponding to overlap ratios of 0.92, 0.84, 0.69, and 0.40 (Fig. 2). Raw diffraction patterns with 256 × 256 pixels were resampled into arrays with 1024 × 1024 pixels before reconstruction. This preprocessing allowed diffraction patterns to be extended to twice the collection angle limit imposed by the detector^26^. The corresponding redundancy parameters σ_pty_^26^ used to evaluate the data with four different overlap ratios were 6.35, 1.61, 0.42, and 0.11, as shown in Fig. 2, with calculation details given in supplemental material I (SM I). For datasets with overlap ratios of 0.92 and 0.84 (the first and second columns in Fig. 2), σ_pty_ is greater than 1, implying that the ptychographic reconstructions are well conditioned^26^. A STEM HAADF^9^ image of the sample is shown in Fig. 1b. This was recorded with the same probe current and a 10μs/pixel dwell time in a 1024 × 1024 pixel array, which corresponded to a dose of 1.35 × 10^5^ e^−^/Å^2^. Projected atomic models of monolayer MoS_2_ crystal structures along <001> are shown in Fig. 1c.

To accurately determine the scanned probe position, which is crucial for high resolution reconstruction, an automatic position refinement algorithm was used^22^ which has previously demonstrated sub-pixel accuracy for electron experiments^34^. An initial guess for the probe function was used for reconstruction, as shown in Fig. S1 and the resulting position refinement is shown in Fig. S2. The MoS_2_ monolayer had a thickness of 0.65 nm, which made it an ideal two-dimensional sample and therefore, the assumption in the ePIE algorithm^43^ that the exit wave can be considered as a product of the probe function and the object transmission function was satisfied.

### Ptychographical reconstructions at various doses

In theory, the resolution in the reconstruction is limited by the highest angle at which electrons can be detected^33,44^. However, because the aim of this work was to evaluate resolution under low-dose conditions, few electrons scattered outside of the bright field (BF) disk could be recorded on the detector, as shown in Fig. S3. Therefore, only the BF disks (α cutoff) were used in the ptychographic reconstructions described in this work.

The four integrated acquisition times and the four overlap ratios described above provided sixteen experimental combinations and their corresponding total doses are summarized in Fig. 2. The corresponding ptychographic phases are shown in Fig. S4. Each row and column of the array of reconstructions illustrates the evolution of the quality of the phase with respect to the integrated acquisition time and the overlap ratio, respectively. It is evident that the visibility of the MoS_2_ lattices decreased with shorter integrated acquisition time or a decreased overlap ratio. To quantify the contrast change as a function of acquisition time and overlap ratio, line profiles for positions across the Mo and 2 S atomic columns were extracted from the reconstructed phases as shown in the first column and row of Fig. S4, respectively. Figure S5a,b show that the phase values at the Mo and 2 S sites decreased as the integrated acquisition time and overlap ratio decreased, respectively. Corresponding simulated results were also calculated as shown in Fig. S6a,b, respectively, which showed a trend consistent with the experimental data in Fig. S5, although the phase values in the simulations are higher than those in the experiment. The simulation parameters are given in SM II. At present the precise source of this is unclear but there has been extensive work on the discrepancy between experiment and simulation for various imaging modes (the Stobbs factor^45–48^) and in general given careful calibration of imaging and detection conditions absolute-scale quantitative imaging is possible. One possible source of the mismatch is provided in previous studies^48^ where comparisons of the experimental and simulated phase of MoS_2,_ reconstructed from 4D STEM datasets show very good agreement assuming a single S layer arising from S loss during acquisition. It is possible that similar damage occurs in our experimental acquisition although we have not observed significant phase variation between different S columns.

**Figure 3:**
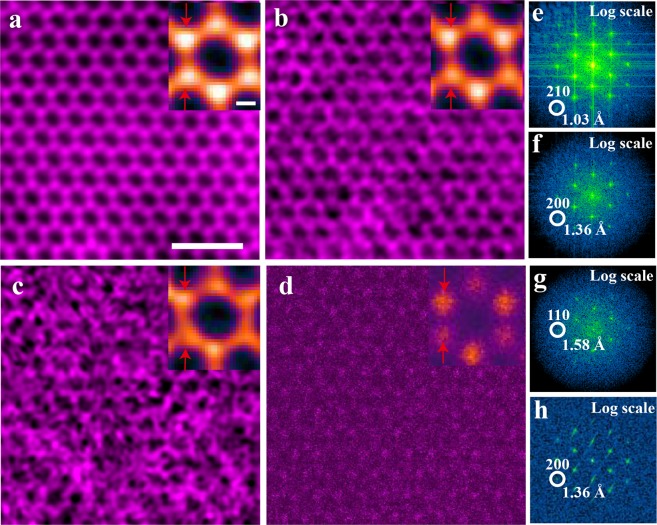
Comparison between phase reconstructions and the HAADF image. (**a**–**c**) Phases of ptychographic reconstructions from the same region with doses of 1.57 × 10^5^, 2.75 × 10^3^ and 4.03 × 10^2^ e^−^/Å^2^; (**d**) HAADF image of monolayer MoS_2_ recorded with a dose of 3 × 10^3^ e^−^/Å^2^. The top-right images inset in (**a**–**d**) are the corresponding experimental unit-cell averaged results, respectively. The 1 nm and 1 Å scale bars apply to (**a**–**d**) and their corresponding insets, respectively. (**e–h)** Power spectra of (**a**–**d**) respectively displayed on a logarithmic intensity scale. Circles indicated (210), (200) and (110) reflections of the MoS_2_ lattice corresponding to spacings of 1.03 Å, 1.36 Å and 1.58 Å.

To quantify the resolution in the reconstructed phases, the corresponding power spectra from each reconstruction in Fig. S4 were calculated, as shown in Fig. S7. The power spectra were calculated from the complex waves of the ptychographic reconstructions, rather than from their phases, since only the complex waves showed linear information transfer. Based on the resolution achieved in each individual image, the array was divided into three regions indicated with different background colors in Fig. 2: high-resolution (HR, 1.03 Å, light blue), mid-resolution (MR, 1.36 Å, blue) and low-resolution (LR, 1.58 Å, dark blue). As a representative result for each resolution region, the reconstructed phases corresponding to doses of 1.57 × 10^5^ e^−^/Å^2^, 2.75 × 10^3^ e^−^/Å^2^, and 4.03 × 10^2^ e^−^/Å^2^ marked by the red squares in Fig. 2 are shown in Fig. 3a–c, respectively.

### Noise robustness in comparison to HAADF imaging

For comparison, a conventional STEM-HAADF (Z-contrast) image^49^ (Fig. 3d) was also acquired with a dose of 3 × 10^3^ e^−^/Å^2^, similar to that in the ptychographic data sets. In the phase, brighter contrast corresponded to a single molybdenum atom (Mo site), and darker contrast to double sulfur atoms (2S site), in MoS_2_ viewed along <001> (Fig. 1c). The top-right insets in Fig. 3a–d are the corresponding experimental unit-cell averaged results. Line profiles extracted from the positions indicated with arrows in the top-right insets are shown in Fig. S8. For an electron dose >10^3^ e^−^/Å^2^, both Mo and 2 S sites in the reconstructed phases (Fig. 3a,b) were clearly resolved and the contrast at the 2S atomic columns was higher, as evident in the line profiles, consistent with the high detection sensitivity of electron ptychography to light elements^34^. In contrast, HAADF imaging at a dose of 10^3^ e^−^/Å^2^ produced a noisy image in which the Mo and 2S atomic sites were not clearly resolved. At the same dose of 10^3^ e^−^/Å^2^, the contrast of the 2S atomic columns in the HAADF image (black curve) is lower than that in the reconstructed phase (blue curve) after normalization with respect to the intensities of the Mo atomic columns as shown in Fig. S8.

To further quantitatively compare the signal to noise behavior of the data at similar dose, two line profiles (Fig. S9) with a width of 3 pixels were extracted across 4 unit cells from the reconstructed phase (Fig. 3b) and HAADF image (Fig. 3d), respectively. The signal to noise ratio (SNR) was then measured as the ratio of the standard deviations of the signal (black curve) and noise (red curve) profiles (Fig. S9) using a method described in ref.^50^ giving the SNR of the reconstructed phase and HAADF images as 11.81 and 0.71. This clearly indicates that defocused probe ptychography is more robust with respect to noise at low dose than conventional HAADF imaging.

To compare resolutions in the reconstructed phases and HAADF images, their corresponding power spectra were calculated and are displayed on a logarithmic scale in Fig. 3e–h. The power spectra (Fig. 3e–g) were calculated from the complex waves of the ptychographic reconstructions (Fig. 3a–c), respectively. Figure 3h shows that the resolution achieved in the HAADF image was 1.36 Å corresponding to the (200) reflections. The same resolution (Fig. 3f) with higher SNR and contrast was obtained in the ptychographic phase at a lower dose than that used in the HAADF image. This demonstrates that the dose efficiency of the ptychographic reconstruction was better than that of HAADF imaging for a 2D material system, such as MoS_2_.

### Dose-dependent spatial resolution

**Figure 4:**
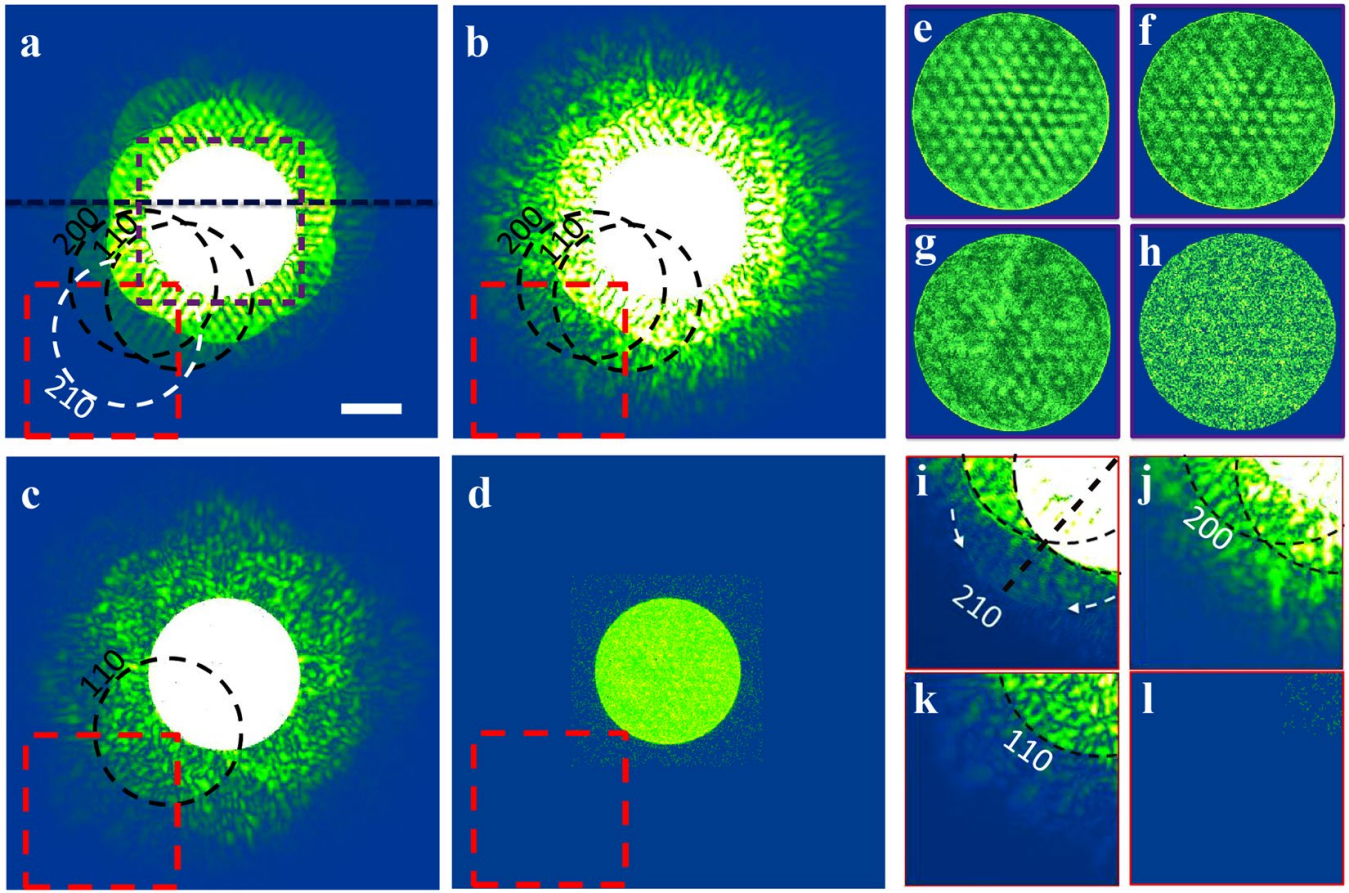
Information strength in the spatial frequency domain. (**a**–**c**) Amplitude of diffraction patterns calculated from the reconstructed phases shown in Fig. 3(a–c), respectively. (**d**) Amplitude of typical raw diffraction pattern acquired at 2 ms. (**e–h**) Enlarged regions extracted from the areas in (**a**–**d**) indicated with a purple square. (**i**–**l)** Enlarged regions extracted from the areas in (**a**–**d**) indicated with a red square. Contrast is boosted to show the low signal reflections (i.e. (210) in (**e**)). The scale bar applied to (**a**–**d**) is 20 mrad.

To evaluate the spatial resolution in object space as shown in Fig. 3a–c, the information strength in the spatial frequency domain was calculated. Figure 4a–c show the amplitude of calculated DPs, obtained by Fourier transforming the product of the reconstructed probe and the reconstructed complex object functions as shown in Fig. 3a–c, respectively. Figure 4d shows the amplitude of a raw DP acquired with a 2 ms exposure that exhibits no high-order reflections. To clearly illustrate the features inside the areas of the BF disk (purple square) and the high order reflections (red square), enlarged images extracted from Fig. 4a–d are shown in Fig. 4e–h and i–l after contrast adjustment. Features in the calculated DPs became increasingly blurred and noisy as the dose decreased.

Line profiles (Fig. S10a) extracted across the DPs at the position indicated by the black line in Fig. 4a, show the evolution of the information retrieved in the calculated DPs as a function of dose. The difference between the values at the peak and valley in the line profiles reduces with decreasing total dose, consistent with reduced information retrieval in the spatial frequency domain. To quantify this variation, the signals within the BF disk were extracted by background subtracting and low band-pass filtering^50^ as shown in Fig. S10b. The standard deviation of the signal at 1.57 × 10^5^ e^−^/Å^2^, for instance is three times of that at 4.03 × 10^2^ e^−^/Å^2^. Similarly, the signal amplitude outside the BF disks (Fig. S10c) shows the same ratio between these two doses. Therefore, the evolution of the information strength in the spatial frequency domain is consistent with our observations in real space as shown in Fig. 3a–c.

In terms of limits to the spatial frequency recovered, higher order reflections from (110), (200) up to (210) were clearly resolved (Fig. 4a) for a dose of 10^5^ e^−^/Å^2^, and gradually faded at lower doses (10^2^ e^−^/Å^2^). The low-contrast, higher order reflection (210), indicated with a dotted circle in Fig. 4a, can be clearly seen in the enlargement shown in Fig. 4i. This suggested that the reconstructed object contained high spatial frequencies due to the (210) reflections and further validated the resolution estimated from the power spectrum (Fig. 3e). The angle of the (210) reflection is nearly twice that of the BF disk (Fig. 4d) in the raw data used in the object reconstruction. This arises since ptychography can effectively extrapolate a diffraction pattern to surpass the aperture (1α cutoff) of the detector, due to the fact that the recorded extent of the diffraction pattern (Fig. 4d) contains information from the spectrum of the specimen up to 2α^26^.

The dose settings for the reconstructions that retrieve the 2α resolution limit imposed by the BF disk (1α cutoff) are shown in the light blue region defined as HR in Fig. 2. In this dose region, no high-resolution information was obtained at doses below 2.04 × 10^4^ e^−^/Å^2^, even though the criterion for dataset redundancy was satisfied (overlap ratio > 0.80 and a redundancy, σ_pty_ >1, defined in SM I)^26^. This implies that a dose threshold (>2.04 × 10^4^ e^−^/Å^2^ in the current configuration) needs to be considered as an additional criterion for retrieval of a 2α resolution limit imposed by a 1α aperture cutoff using iterative ptychography. Overall, at high electron dose, the resolution is determined by the maximum detector angle and the degree of redundancy of the dataset, whereas at low dose Poisson noise is dominant^32^.

This dose-dependence can be further observed in the MR region (blue, 1.36 Å) in Fig. 2, where the resolution is not directly related to the degree of redundancy (overlap ratio or σ_pty_) in the dataset. As an example, although the redundancy in the first column of Fig. 2 (σ_pty_ = 6.35) for a dose of 1.97 × 10^4^ e^−^/Å^2^ was higher than that in the third column of Fig. 2 (σ_pty_ = 0.42) for a dose of 1.10 × 10^4^ e^−^/Å^2^, the resultant reconstructed resolutions were the same. In iterative ptychography, the degree of redundancy in a ptychographical dataset can be estimated as the ratio of the total pixels of known (DPs) and unknown (probe and object) functions (SM I)^26^. As the dose decreases, the influence of Poisson noise becomes important as there are insufficient electrons scattered into each pixel to provide interference in the DPs. As a result, the total number of useable pixels in the known functions (DPs) is reduced and hence the effective degree of redundancy decreases. Therefore, ultimately in the low dose regime the resolution is dose limited.

Finally, we calculated the root-mean-square width of the Mo atomic columns measured from the standard deviation of a Gaussian fit as a function of the dose as shown in Fig. S11. In comparison to simulation data reproduced from Fig. 5b in Jiang *et al*.^32^, the resolution of our experimental results show a similar trend with respect to dose. However, the experimental resolution is slightly larger than that in the simulations by 0.02–0.1 Å due to experimental instabilities (i.e. sample and thermal drift and noise in the deflector currents).

### High resolution reconstructions at low dose

In the LR region (dark blue) in Fig. 2, the total dose was experimentally decreased to a value of 403 e^−^/Å^2^, at which lattice contrast was still visible in the reconstructed phase (Fig. 3c) and the power spectrum (Fig. 3g) showed strong (110) reflections corresponding to a 1.58 Å spacing. This result is consistent with the simulations at low dose reported by Müller *et al*.^32^. Notably, previous experimental reports of defocused ptychographic reconstructions at atomic resolution had significantly higher electron doses. As examples, Putkunz *et al*.^36^, D’Alfonso *et al*.^30^, and Wang *et al*.^34^ imaged nanostructured crystals at a dose of 10^6^ e^−^/Å^2^, whereas Humphry *et al*.^33^ used a dose of 1273 e^−^/Å^2 30^, which is three times that reported here, for a maximum resolution of 2.36 Å in their reconstruction.

Recent work by Müller *et al*.^32^ has shown that an experimental reconstruction can achieve a resolution of 0.4 Å, at a dose of 1.8 × 10^5^ e^−^/Å^2^. To perform focused probe ptychography at low dose (i.e. 500 e^−^/Å^2^), the beam current used can be theoretically estimated to be around 0.1–0.01 pA, which is significantly lower than typical beam currents (1–50 pA) used for STEM imaging^32^. This may lead to significant difficulties in corrector alignment and sample region location. In contrast, the beam current for defocused probe ptychography used in this work was 17 pA, similar to typical operating conditions. Therefore, defocused ptychography not only facilitates easier practical operation, but also provides potential to reach a dose suitable for phase retrieval from biological materials^35^ at lower resolution than that reported here.

Overall, the resolution achieved in the low-dose reconstructed phase could be attributed to several factors. Compared to HAADF imaging, ptychography uses the whole of the BF disk within which are the majority of scattered electrons, thereby increasing the dose efficiency. Secondly, the reconstruction algorithm used was robust with respect to noise and also acted as an effective noise filter^43^. Thirdly, the use of a direct-detection counting camera enabled fast collection of low signals with a high SNR.

Finally, at low electron dose, defocused probe ptychography shows better reconstruction quality^32^ and is less reliant on extremely low beam currents than the focused probe geometry. Thus, this method has the potential to provide quantitative phase information from beam-sensitive samples such as organic crystals^51^, biological materials^12^ and frozen specimens^35^ at high resolution without damage.

## Conclusions

Using a direct detection camera, resolution in the phase of ptychographic reconstructions of a MoS_2_ monolayer has been evaluated under low electron dose conditions by varying the DP acquisition time and the overlap ratio. For high resolution, low dose imaging, it is essential to ensure that all electrons incident on the sample contribute to the final image. Compared to HAADF imaging using high angle scattered electrons, which form a small portion of the total incident electrons, the phase of the ptychographic reconstruction is more efficient and provides reconstructed data with better contrast and SNR for a similar electron dose budget. However, extending the resolution to twice that defined by the BF disk required a relatively high dose condition (>2.04 × 10^4^ e^−^/Å^2^), although a reconstruction with a 1.58 Å resolution was observed at an electron dose of 403 e^−^/Å^2^. Finally, the parameters reported here should provide guidelines for ptychography of beam-sensitive samples including biological materials.

## Supplementary information

supplementary information

## References

[1] Haider M (1998). Electron microscopy image enhanced. Nature.

[2] Urban K, Kabius B, Haider M, Rose H (1999). A way to higher resolution: spherical-aberration correction in a 200 kV transmission electron microscope. J. Electron Microsc..

[3] Schmidt T (2010). Double aberration correction in a low-energy electron microscope. Ultramicroscopy.

[4] Kabius B (2009). Special number: Instrumentation/Performance First application of Cc-corrected imaging for high-resolution and energy-filtered TEM. J. Electron Microsc..

[5] Krivanek OL (2009). High-energy-resolution monochromator for aberration-corrected scanning transmission electron microscopy/electron energy-loss spectroscopy. Phil. Trans. Roy. Soc Series A.

[6] Muller DA (2009). Structure and bonding at the atomic scale by scanning transmission electron microscopy. Nat. Mater..

[7] Kaiser U (2011). Transmission electron microscopy at 20 kV for imaging and spectroscopy. Ultramicroscopy.

[8] Huang WJ, Zuo JM, Jiang B, Kwon KW, Shim M (2008). Sub-ångström-resolution diffractive imaging of single nanocrystals. Nat. Phys..

[9] Sawada H (2009). STEM imaging of 47-pm-separated atomic columns by a spherical aberration-corrected electron microscope with a 300-kV cold field emission gun. J. Electron Microsc..

[10] Egerton RF, Li P, Malac M (2004). Radiation damage in the TEM and SEM. Micron.

[11] Gibson JB, Goland AN, Milgram M (1987). & Vineyard, G. H. Dynamics of Radiation Damage. Phys. Rev..

[12] Glaeser RM (1971). Limitations to significant information in biological electron microscopy as a result of radiation damage. J. Ultrastructure Res..

[13] Yoshida K, Sasaki Y (2012). Optimal accelerating voltage for HRTEM imaging of zeolite. J. Electron Microsc..

[14] Hobbs LW (1979). Application of Transmission Electron Microscopy to Radiation Damage in Ceramics. J. Am. Ceramic Soc..

[15] Grubb DT (1974). Radiation damage and electron microscopy of organic polymers. J. Mat. Sci..

[16] Buban JP, Ramasse Q, Gipson B, Browning ND, Stahlberg H (2010). High-resolution low-dose scanning transmission electron microscopy. J. Electron Microsc..

[17] Kramberger C, Mittelberger A, Hofer C, Meyer JC (2017). Analysis of Point Defects in Graphene Using Low Dose Scanning Transmission Electron Microscopy Imaging and Maximum Likelihood Reconstruction. Phys. Status Solidi B.

[18] Mittelberger A, Kramberger C, Hofer C, Mangler C, Meyer JC (2017). Automated Image Acquisition for Low-Dose STEM at Atomic Resolution. Micros. & Microanal..

[19] Mittelberger A, Kramberger C, Meyer JC (2018). Software electron counting for low-dose scanning transmission electron microscopy. Ultramicroscopy.

[20] Vila-Comamala J (2011). Characterization of high-resolution diffractive X-ray optics by ptychographic coherent diffractive imaging. Opt. Express.

[21] Zheng G, Nanda P, Shiradkar R, Dong S (2014). Spectral multiplexing and coherent-state decomposition in Fourier ptychographic imaging. Biomedical Opt. Express.

[22] Zhang F (2013). Translation position determination in ptychographic coherent diffraction imaging. Opt. Express.

[23] De Caro L, Carlino E, Caputo G, Cozzoli PD, Giannini C (2010). Electron diffractive imaging of oxygen atoms in nanocrystals at sub-angstrom resolution. Nat. Nano..

[24] Zuo JM, Vartanyants I, Gao M, Zhang R, Nagahara LA (2003). Atomic resolution imaging of a carbon nanotube from diffraction intensities. Science.

[25] Weierstall U (2001). Image reconstruction from electron and X-ray diffraction patterns using iterative algorithms: experiment and simulation. Ultramicroscopy.

[26] Maiden AM, Humphry MJ, Zhang F, Rodenburg JM (2011). Superresolution imaging via ptychography. J. Opt. Soc. Am. A.

[27] Nellist PD, Mccallum BC, Rodenburg JM (1995). Resolution beyond the ‘information limit’ in transmission electron microscopy. Nature.

[28] Pennycook TJ (2015). Efficient phase contrast imaging in STEM using a pixelated detector. Part 1: experimental demonstration at atomic resolution. Ultramicroscopy.

[29] Gao S (2017). Electron ptychographic microscopy for three-dimensional imaging. Nat. Commun..

[30] D’Alfonso AJ, Allen LJ, Sawada H, Kirkland AI (2016). Dose-dependent high-resolution electron ptychography. J. Appl. Phys..

[31] Yang H (2016). Simultaneous atomic-resolution electron ptychography and Z-contrast imaging of light and heavy elements in complex nanostructures. Nat. Commun..

[32] Jiang Y (2018). Electron ptychography of 2D materials to deep sub-ångström resolution. Nature.

[33] Humphry MJ, Kraus B, Hurst AC, Maiden AM, Rodenburg JM (2012). Ptychographic electron microscopy using high-angle dark-field scattering for sub-nanometre resolution imaging. Nat. Commun..

[34] Wang P, Zhang F, Gao S, Zhang M, Kirkland AI (2017). Electron Ptychographic Diffractive Imaging of Boron Atoms in LaB6Crystals. Sci. Rep..

[35] Pelz, P. M., Qiu, W. X., Bücker, R., Kassier, G. & Miller, R. J. D. Low-dose cryo electron ptychography via non-convex Bayesian optimization. *Sci. Rep*. **7** (2017). 10.1038/s41598-017-07488-y PMC5575234 28851880

[36] Putkunz CT (2012). Atom-scale ptychographic electron diffractive imaging of boron nitride cones. Phys. Rev. Letts..

[37] D’Alfonso, A. J. *et al*. Deterministic electron ptychography at atomic resolution. *Phys. Rev. B* **89** (2014).

[38] Mir JA (2017). Characterisation of the Medipix3 detector for 60 and 80keV electrons. Ultramicroscopy.

[39] Tate MW (2016). High Dynamic Range Pixel Array Detector for Scanning Transmission Electron Microscopy. Micros. & Microanal..

[40] Sawada H (2014). Super High Resolution Imaging with Atomic Resolution Electron Microscope of JEM-ARM300F[J]. JEOL News.

[41] Hue F, Rodenburg JM, Maiden AM, Midgley PA (2011). Extended ptychography in the transmission electron microscope: possibilities and limitations. Ultramicroscopy.

[42] Song J (2018). Fast and Low-dose Electron Ptychography. Micros. & Microanal..

[43] Maiden AM, Rodenburg JM (2009). An improved ptychographical phase retrieval algorithm for diffractive imaging. Ultramicroscopy.

[44] Gabor, D. A new microscope principle. *Nature* **161** (1948). 10.1038/161777a0 18860291

[45] LeBeau JM, Findlay SD, Allen LJ, Stemmer S (2008). Quantitative atomic resolution scanning transmission electron microscopy. Phys. Rev. Letts..

[46] Thust A (2009). High-resolution transmission electron microscopy on an absolute contrast scale. Phys. Rev. Letts..

[47] LeBeau, J. M., D’Alfonso, A. J., Findlay, S. D., Stemmer, S. & Allen, L. J. Quantitative comparisons of contrast in experimental and simulated bright-field scanning transmission electron microscopy images. *Phys. Rev. B* **80** (2009).

[48] Chen Z (2016). Practical aspects of diffractive imaging using an atomic-scale coherent electron probe. Ultramicroscopy.

[49] Nellist PD (2004). Direct Sub-Angstrom Imaging of a Crystal Lattice. Science.

[50] Jones L, Nellist PD (2013). Identifying and correcting scan noise and drift in the scanning transmission electron microscope. Micros. & Microanal..

[51] Harano K (2012). Heterogeneous nucleation of organic crystals mediated by single-molecule templates. Nat. Mater..

