# Supplementary material for: Atomic Resolution Defocused Electron Ptychography at Low Dose with a Fast, Direct Electron Detector
Source: Sci Rep. 2019 Mar 8;9:3919. doi: 10.1038/s41598-019-40413-z (PMC6408533; doi:10.1038/s41598-019-40413-z)
Supplement: Supplementary file 1 — supplementary information [file 41598_2019_40413_MOESM1_ESM.pdf]

Supplementary Material

Atomic Resolution Defocused Electron Ptychography at Low Dose with a Fast, Direct Electron Detector

Jiamei Song[1], Christopher S. Allen[2,3], Si Gao[1], Chen Huang[2,3], Hidetaka Sawada[4], Xiaoqing Pan[5], Jamie Warner[2], Peng Wang[1*] and Angus I. Kirkland[2,3]

[1] *College of Engineering and Applied Sciences, Nanjing University, Nanjing 210093, People's Republic of China.*

[2] *Department of Materials, University of Oxford, Parks Road, Oxford OX1 3PH, UK.*

[3] *Electron Physical Sciences Imaging Centre, Diamond Lightsource Ltd., OX11 0DE, U.K.*

[4] *JEOL Ltd, 1-2 Mushashino, 3-Chome, Akishima, Tokyo 196, Japan.*

[5] *Department of Materials Science and Engineering, and Department of Physics and Astronomy, University of California, Irvine, CA 92697, USA.*

*Correspondence to: wangpeng@nju.edu.cn

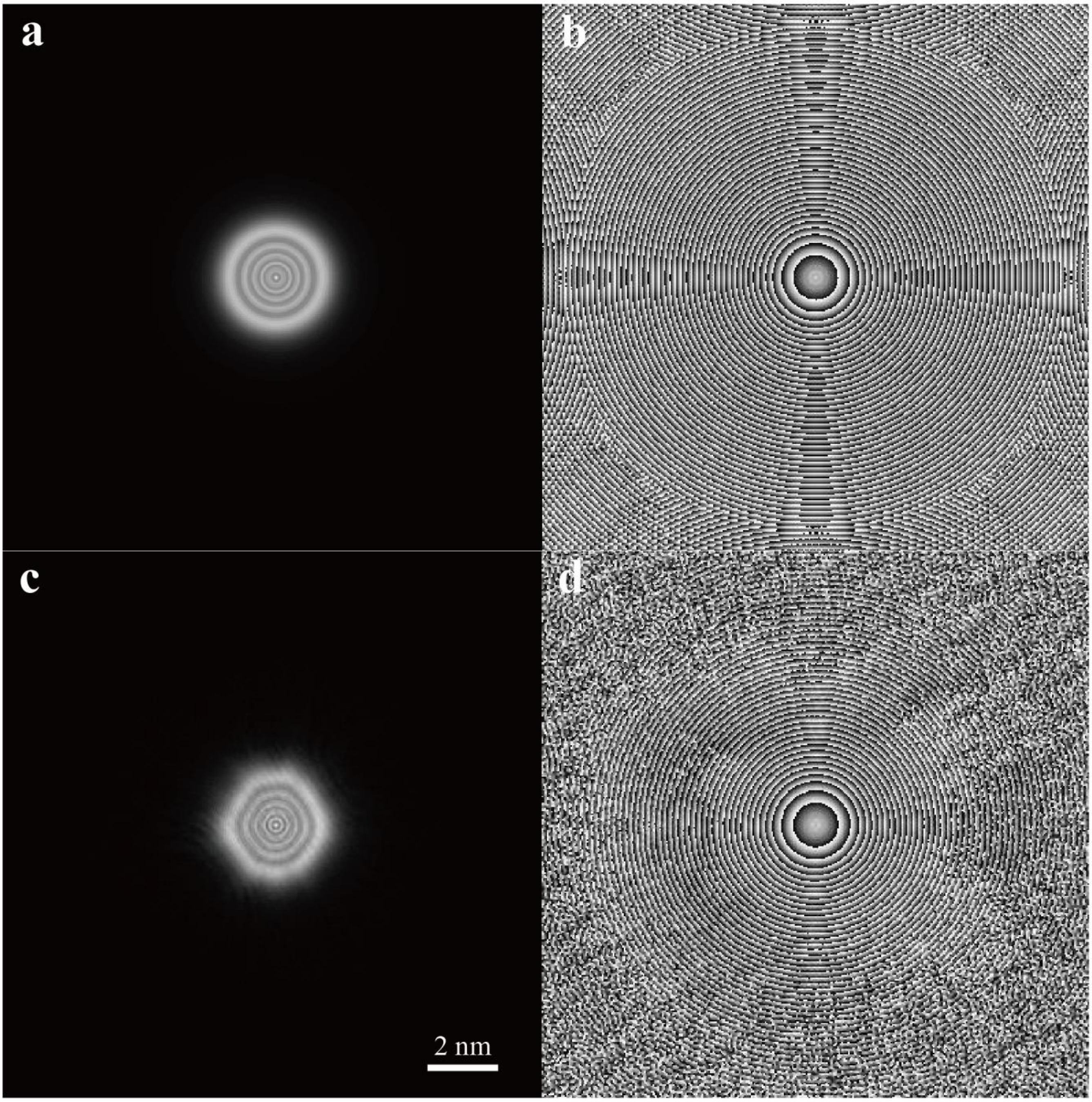

Figure S1. Estimated and reconstructed probe functions. a, Modulus and **b**, Phase of the initial estimated probe function at defocus = -80 nm. **c,** Modulus and **d,** Phase of the probe function after reconstruction. The hexagonal symmetry in the reconstructed probe arises from uncorrected residual higher order aberrations.

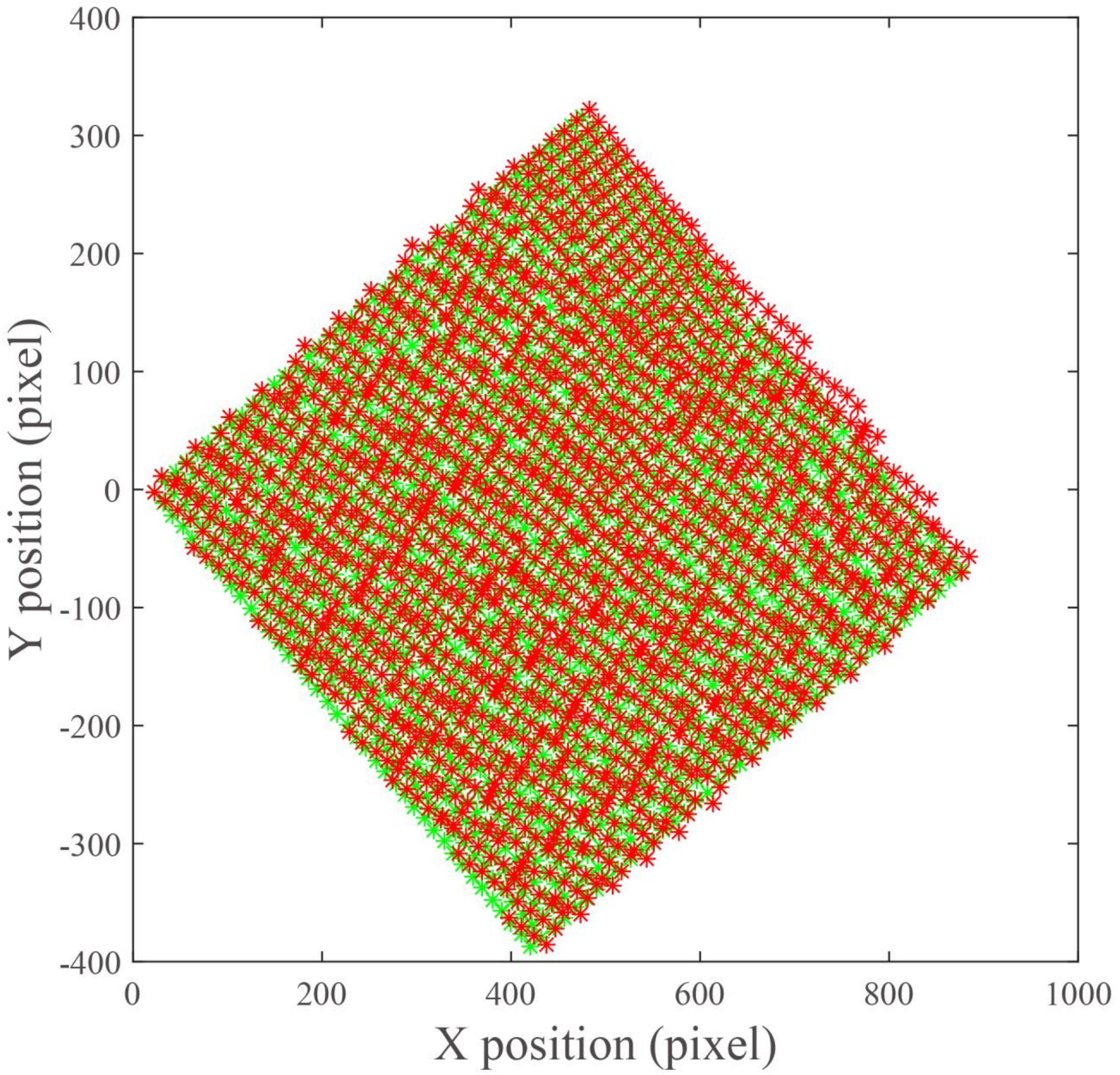

Figure S2. Comparison of positions between nominal X-Y scans (green *) and retrieved probe positions (red *) used in the reconstruction of the region shown in Fig. 3(a-c). The deviation was mainly caused by hysteresis in the scanning coils.

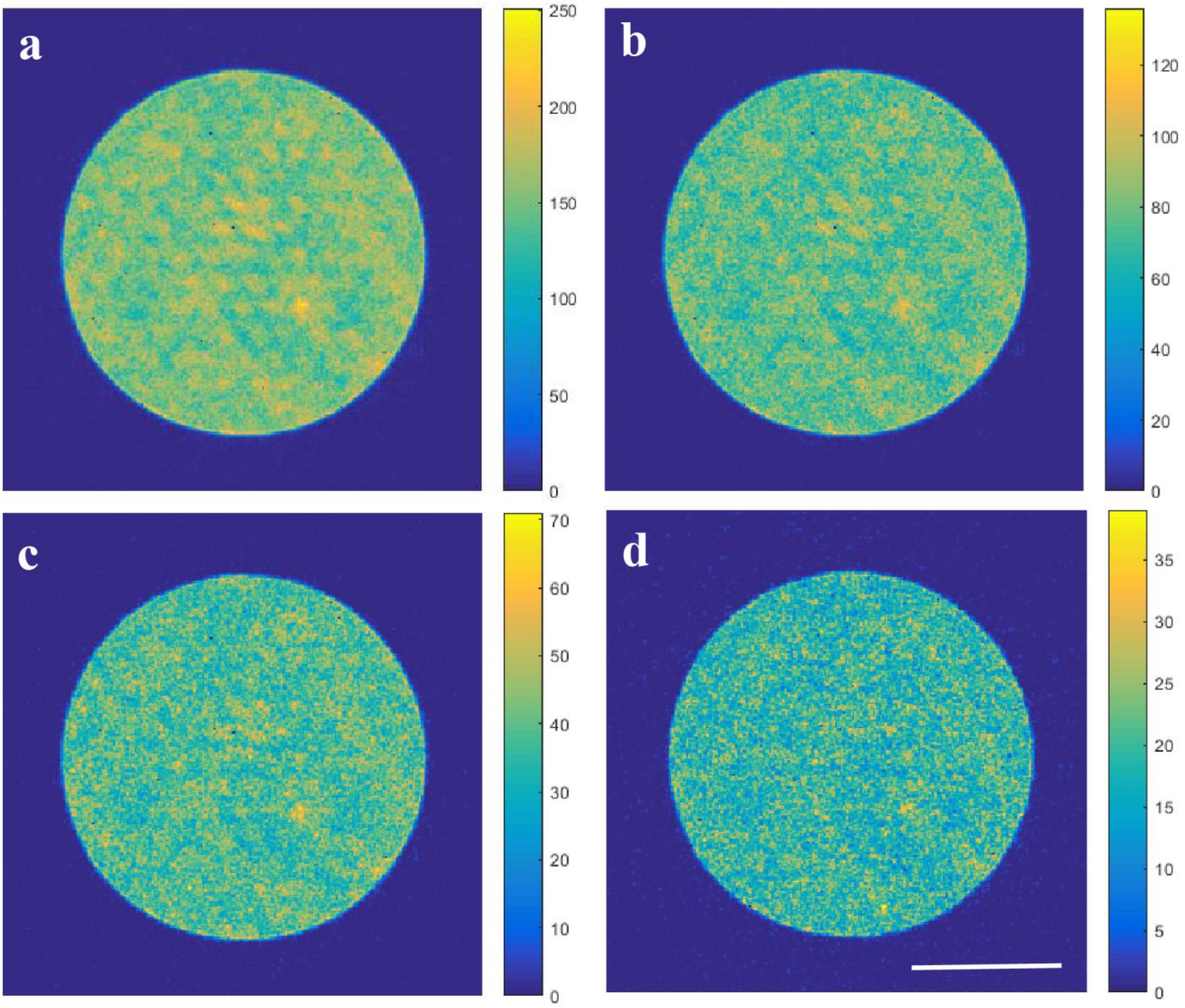

Figure S3. Typical raw diffraction patterns with different acquisition times. Raw diffraction patterns with integrated acquisition times of **a,** 16 ms, **b,** 8 ms, **c,** 4 ms, **d,** 2 ms. Few electrons were scattered outside the bright field disk. The scale bar is 20 mrad.

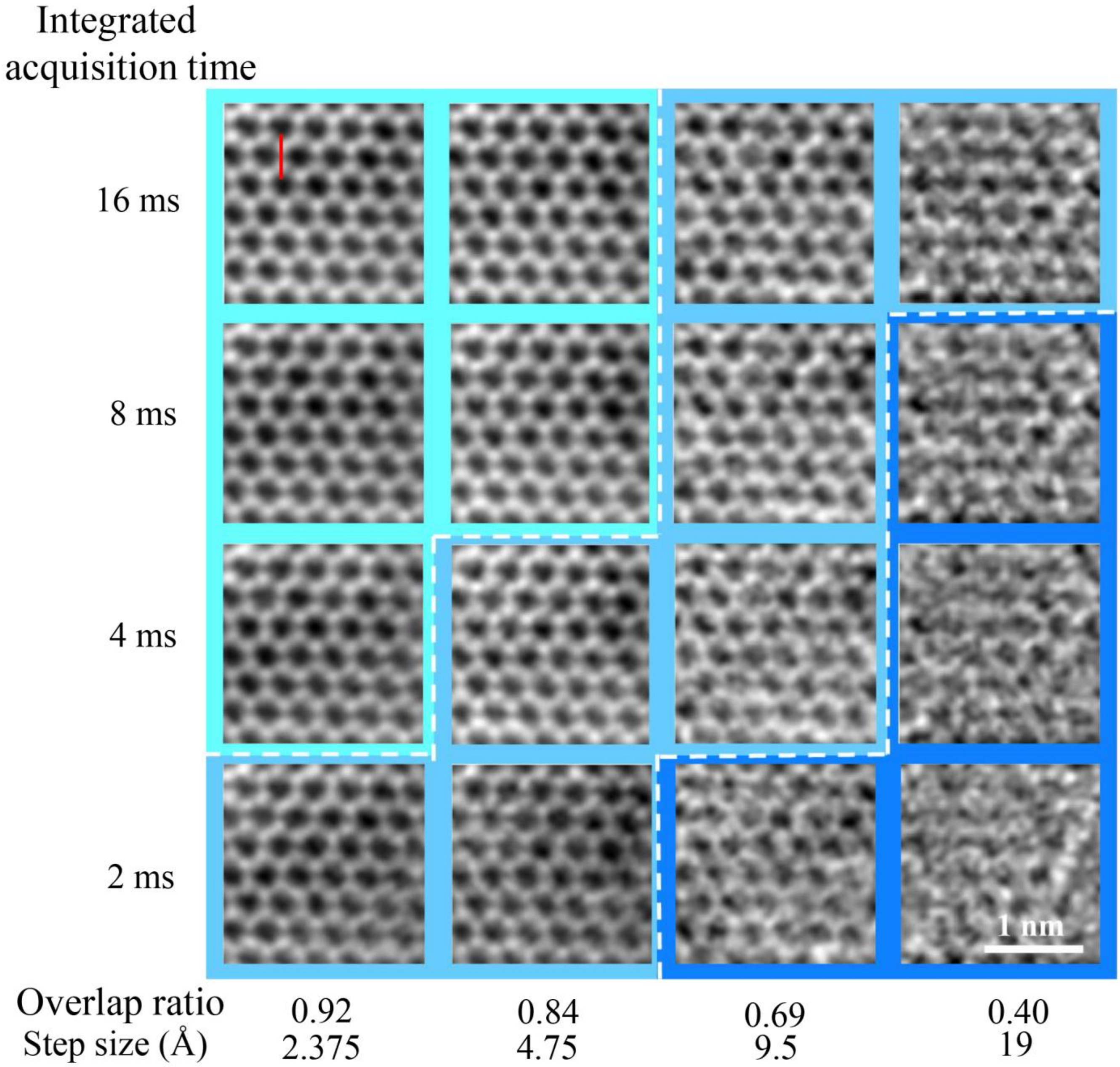

Figure S4. Phases of the ptychographic reconstruction for different acquisition times and overlap ratios with a probe-forming convergence semi-angle of 24 mrad at an acceleration voltage of 80 kV. The corresponding electron doses are listed in Fig. 2. Line profiles shown in Fig. S6 were extracted from the reconstructed phases as indicated by the red line upper left. The scale bar is 1 nm.

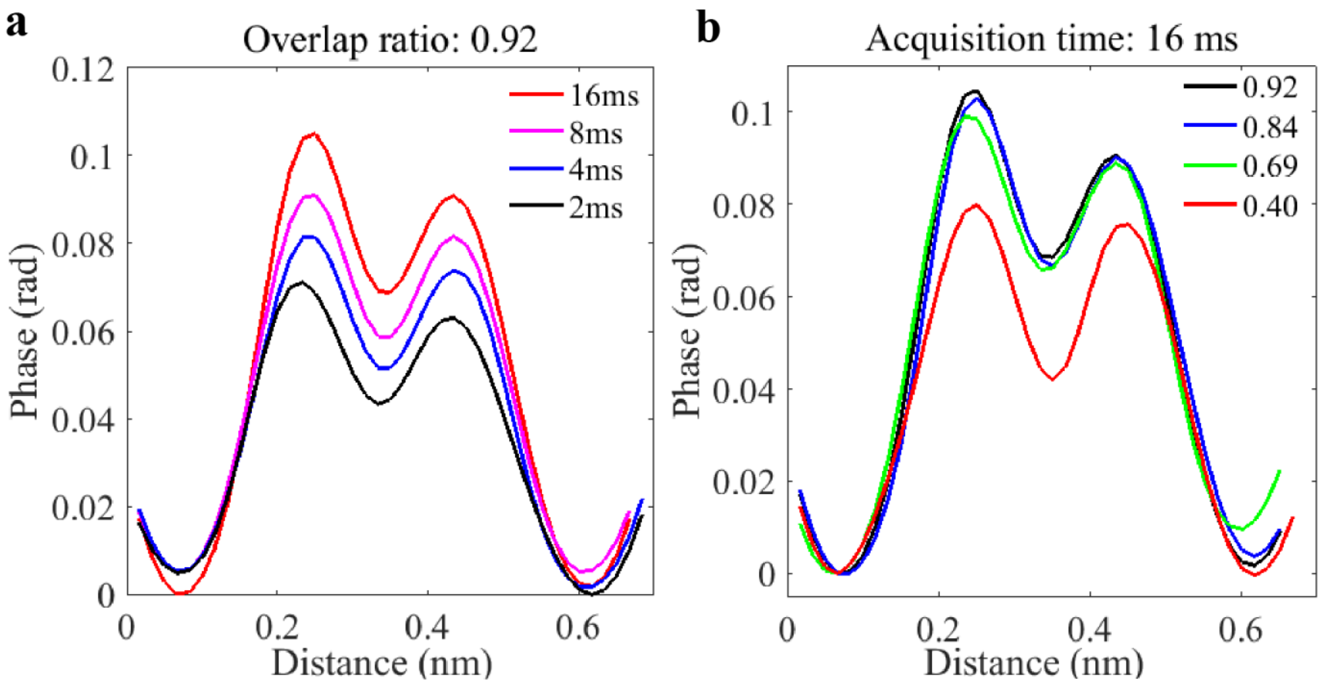

Figure S5. Averaged line profiles with a width of 3 pixels extracted at the position of the red line shown in Fig. S4. a, Line profiles for different acquisition times but the same overlap ratio. **b,** Line profiles for different overlap ratios but the same acquisition time.

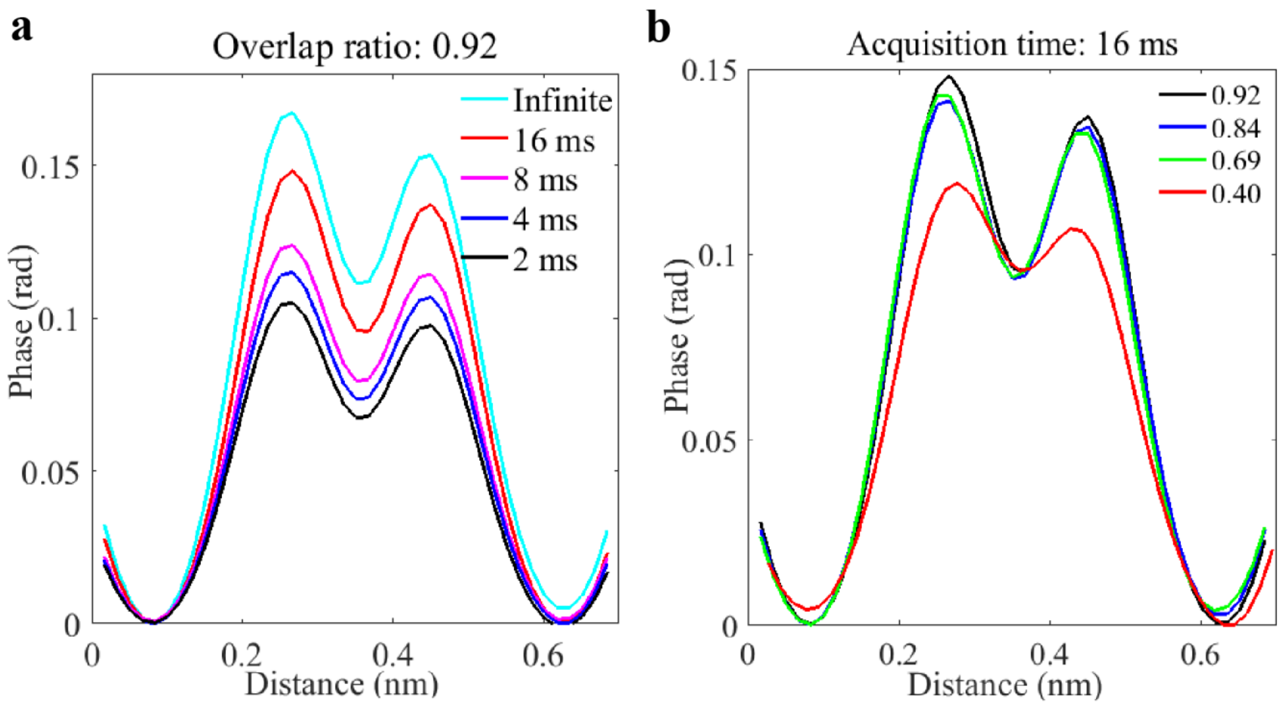

Figure S6. Average line profiles with a width of 3 pixels extracted from simulated phases. a, Line profiles for different acquisition times and constant overlap ratio 0.92. **b,** Line profiles for different overlap ratios and constant acquisition time of 16 ms.

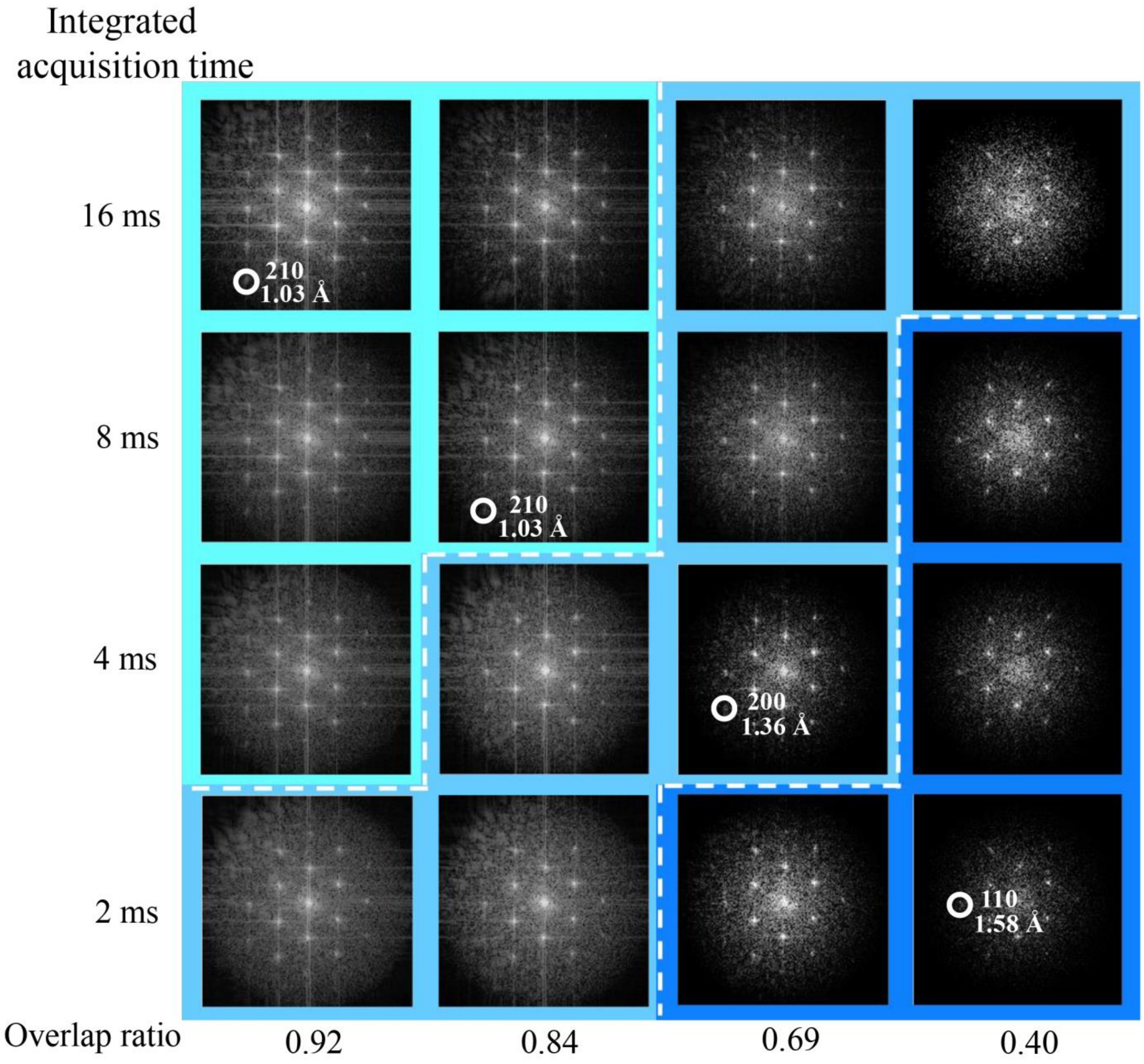

Figure S7. Power spectra of the reconstructed object function for different acquisition times and overlap ratios displayed on a logarithmic intensity scale. The light blue, blue and dark blue backgrounds correspond to high-resolution (HR, 1.03 Å), mid-resolution (MR, 1.36 Å) and low-resolution (LR, 1.58 Å), respectively as in Fig. 2.

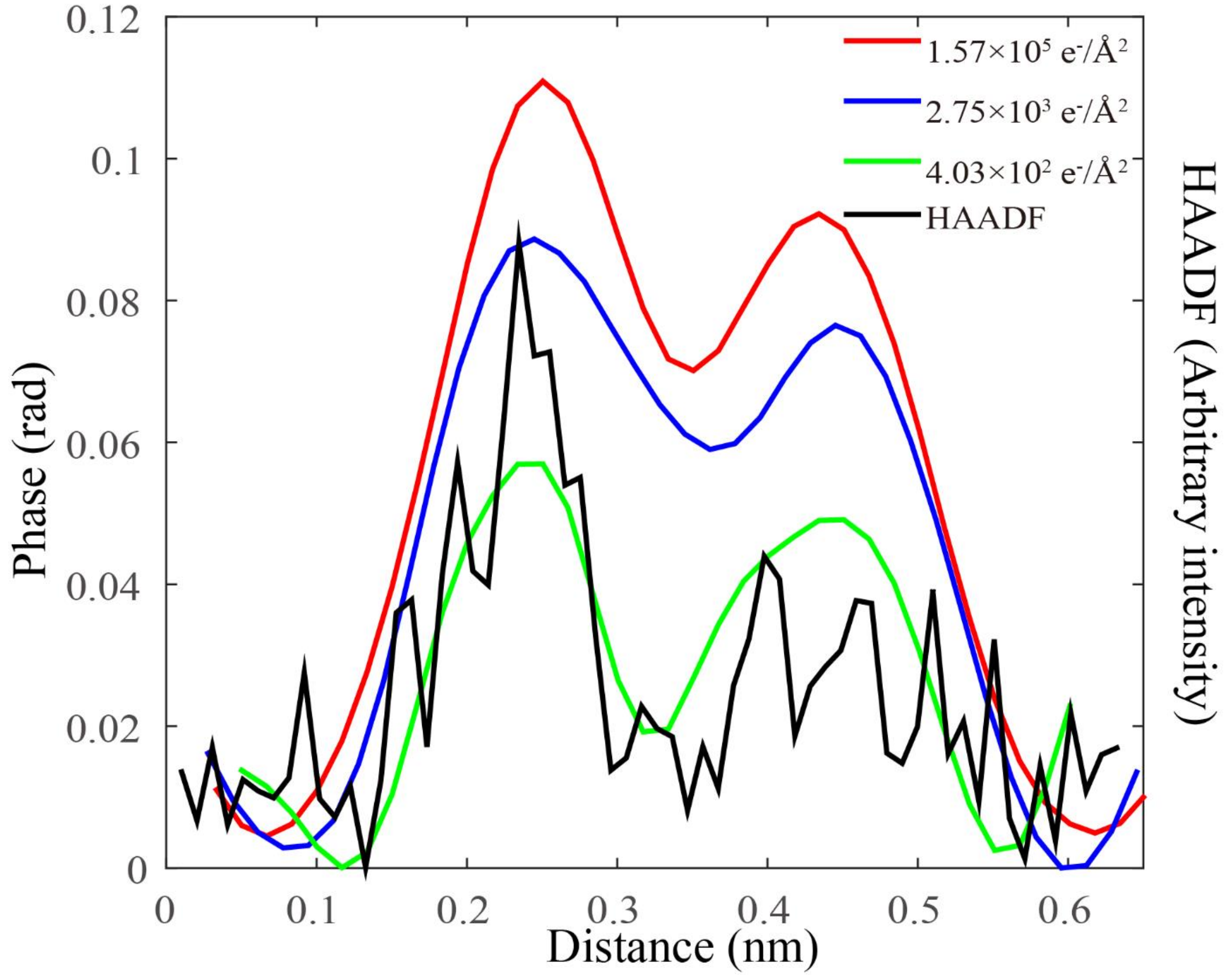

Figure S8. Line profiles with a width of 3 pixels extracted from the top-right insets in the reconstructed phases and the HAADF image in Fig. 3(a-d), respectively.

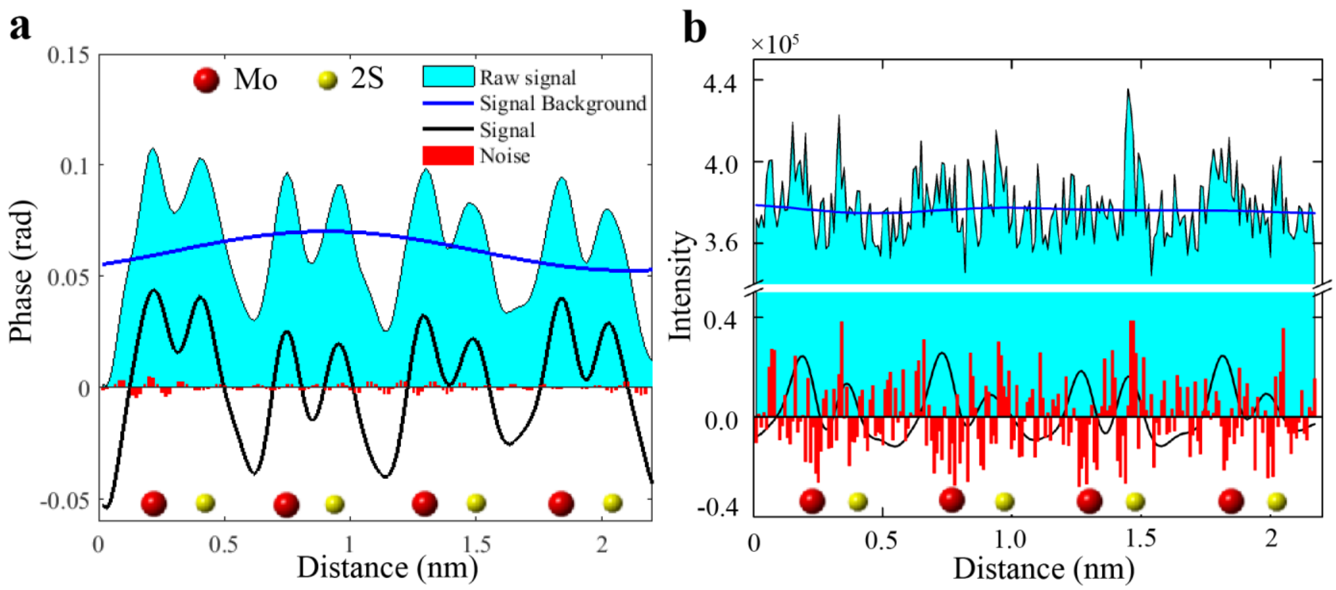

Figure S9. Line profiles extracted from the reconstructed phase and the HAADF image under similar dose. Line profiles (cyan) extracted from **a,** the reconstructed phase in Fig. 3b and **b,** the HAADF image in Fig. 3d. The components are divided into signal background (blue line), signal (black line), noise (red bars).

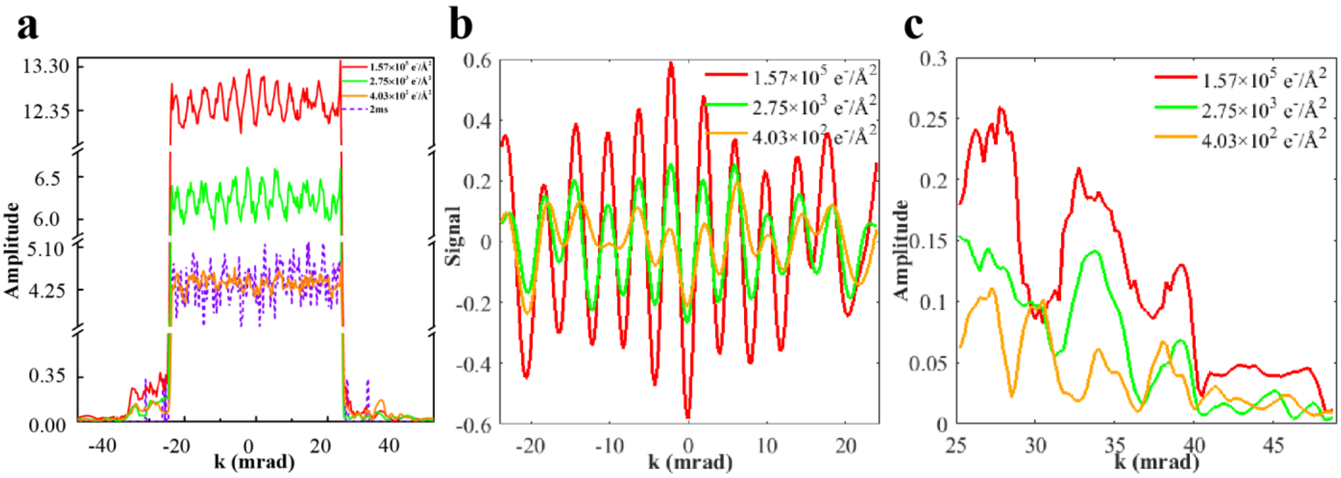

Figure S10. Line profiles showing the evolution of the information retrieved in the calculated DPs as a function of the dose. a, Line profiles with a width of 3 pixels extracted across the diffraction patterns in Fig. 4 (a-d) indicated with the black line in Fig. 4a. **b,** Background subtracted signal extracted from the line profiles inside of the BF disk in (a) by applying a low band-pass filter. **c,** Enlarged line profiles of the amplitude with a width of 3 pixels extracted outside of the BF disk from 25 mrad to 48 mrad indicated with the black line in Fig. 4i.

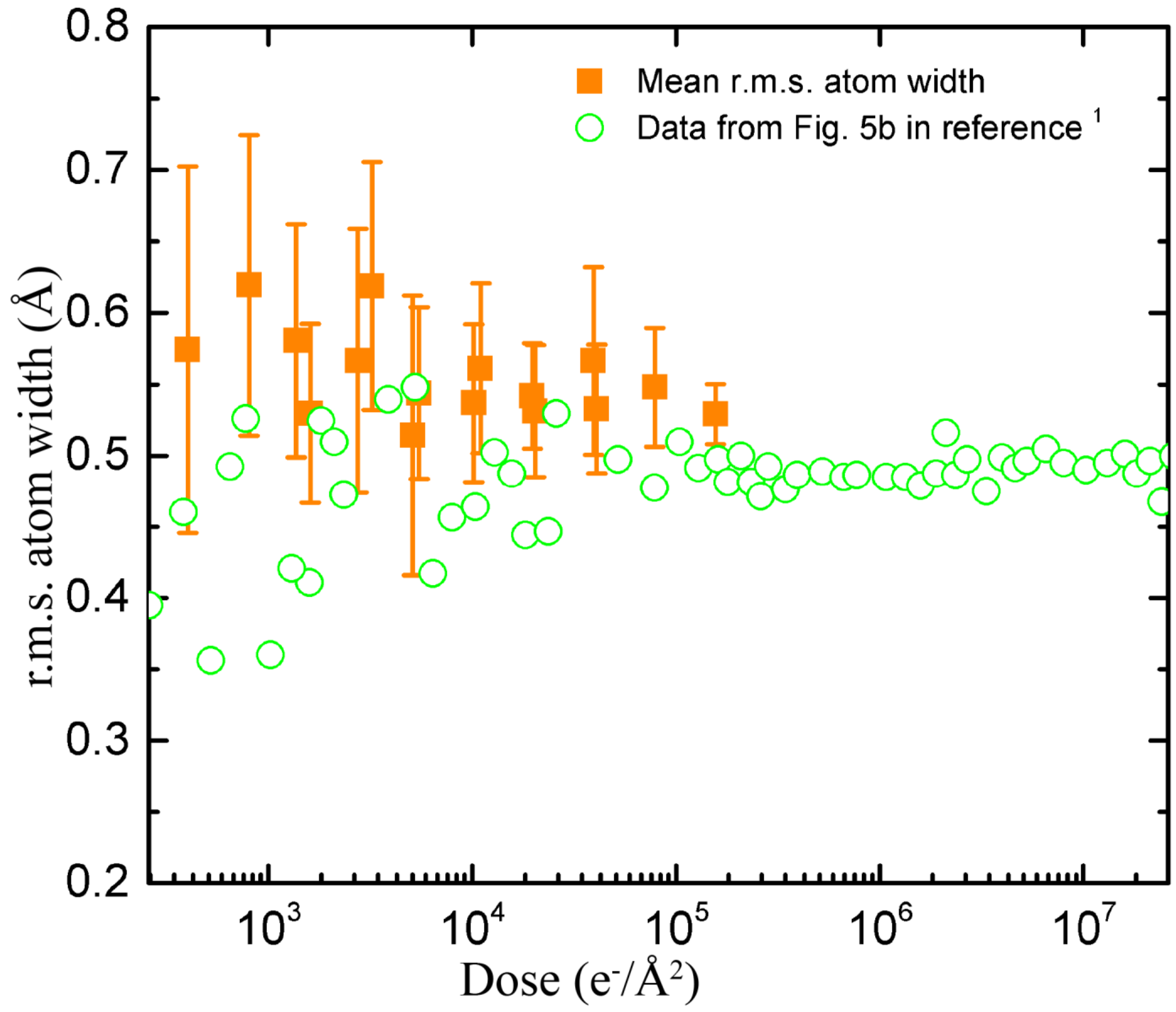

Figure S11. A comparison of the root-mean-square (r.m.s.) width of the molybdenum atom columns in Fig. S4 and from Fig. 5b in Ref [1] measured from the standard deviation of a Gaussian fit as a function of the dose.

TABLE S1. Parameters for ePIE reconstructions using varying step sizes. σ_{pty} is the parameter used to evaluate the degree of redundancy of ptychographical dataset [2] (See SM I for the details of the evaluation).

Step size(Å)	Numbers of diffraction pattern	Overlap ratio	Pixels in reconstruction	Pixels in specimen	σ_{pty}
2.375	40×40	0.92	193^2	1832×1988	6.35
4.75	20×20	0.84	193^2	1815×1966	1.61
9.5	10×10	0.69	193^2	1784×1912	0.42
19	5×5	0.40	193^2	1704×1825	0.11

I. The parameter σ_{pty} in Supplementary Table 1 used to evaluate the degree of redundancy of dataset

In ptychography, σ_{pty}^2 can be used to estimate the degree of redundancy in a dataset, related to an over-sampling ratio as:

$$\sigma_{pty} = \frac{J(\text{pixels per diffraction pattern})}{2(\text{pixels in specimen \& probe reconstructions})} \quad (1)$$

where J represents the total number of pixels in the DPs used for reconstruction. Taking the dataset in the first column of Fig. S4 as an example, the reconstructed object consisted of 1832 × 1988 pixels. Each diffraction pattern only used central 193 × 193 pixels in the iterative reconstruction. The number of recorded DPs is 40 × 40. Therefore, the total number of pixels in the DPs used for reconstruction (J) is 1600 × 193^2, while the number of unknown pixels in the specimen and probe was 2 × (1833 × 1988 + 1024^2). σ_{pty} is hence evaluated as 6.35, implying that the ptychographic reconstruction is well conditioned for the dataset in the first column in Fig. S4. Parameters for reconstructions using varying step sizes are listed in Supplementary Table 1.

II. Multislice Simulation of DPs for Ptychography

To confirm the interpretation of our experimental results, we have carried out a ptychographic reconstruction from an array of simulated diffraction patterns calculated using the multislice method[3] using code due to Kirkland[4]. A model specimen was constructed as an <001> oriented monolayer MoS_2 crystal with a thickness of 3.17Å as shown in Fig.1c. The incident electron energy was 80 kV and the convergence semi-angle was 24 mrad. A simulated array of 40×40 diffraction patterns (DPs) were calculated with each pattern calculated on a 1024 ×1024 pixel array with a sampling[5,6] of 0.25 mrad/pixel. A 256 × 256 mask was subsequently added to the center of each pattern to exclude outer signals. The scanning step size was 0.23 nm. The "frozen phonon" method was used in the multislice simulation with 20 phonon configurations for each simulated DP. Including frozen phonon states the calculated intensity of diffraction patterns was, therefore, an incoherent superposition of the images formed for each atomic configuration over the range of atomic positions given by the Debye-Waller factors (0.093354Å and 0.1678854Å for Mo and S atoms, respectively[7-9]). Poisson distributed shot noise corresponding to the experimental dose conditions was subsequently added to each diffraction patterns using code written in Matlab [10,11]. The phases were subsequently recovered using ePIE[12,13] with different overlap ratios and counts corresponding to experimental datasets with exposure times of 16 ms, 8 ms, 4ms and 2 ms and then convoluted with a Gaussian function with a full width at half maximum (FWHM) of 0.09nm to model the partial coherence arising from the finite extent of the effective electron source[14].

Supplementary References:

1. Jiang, Y. *et al.* Deep sub-Ångstrom imaging of 2D materials with a high dynamic range detector. *Nature* **559**, 343 (2018).
2. Maiden, A. M., Humphry, M. J., Zhang, F. & Rodenburg, J. M. Superresolution imaging via ptychography. *J. Opt. Soc. Am. A* **28**, 604-612 (2011).
3. Cowley, J. M. & Moodie, A. F. The scattering of electrons by atoms and crystals. I. A new theoretical approach. *Acta Crystallogr.* **10**, 609-619 (1957).
4. Kirkland, E. J. *Advanced Computing in Electron Microscopy*. (Springer US, 2010).
5. Batey, D. J. *et al.* Reciprocal-space up-sampling from real-space oversampling in x-ray ptychography. *Phys. Rev. A* **89** (2014).
6. Edo, T. B. *et al.* Sampling in x-ray ptychography. *Phys. Rev. A* **87** (2013).
7. Peng, L. M. Electron atomic scattering factors, Debye-Waller factors and the optical potential for high-energy electron diffraction. *J. Electron Microsc.* **54**, 199-207 (2005).
8. Loane, R. F., Xu, P. & Silcox, J. Thermal vibrations in convergent-beam electron diffraction. *Acta Cryst. A* **47**, 267 (1991).
9. Gao, H. X. & Peng, L. M. Parameterization of the temperature dependence of the Debye-Waller factors. *Acta Cryst. A: Foundations of Crystallography* **55**, 926-932 (2010).
10. Huang., X. *et al.* Signal-to-noise and radiation exposure considerations in conventional and diffraction x-ray microscopy. *Opt. Express* **17**, 13541 (2009).
11. Godard, P., Allain, M., Chamard & V. & Rodenburg, J. Noise models for low counting rate coherent diffraction imaging. *Opt. Express* **20**, 25914-25934 (2012).
12. Hue, F., Rodenburg, J. M., Maiden, A. M. & Midgley, P. A. Extended ptychography in the transmission electron microscope: possibilities and limitations. *Ultramicroscopy* **111**, 1117-1123 (2011).
13. Maiden, A. M. & Rodenburg, J. M. An improved ptychographical phase retrieval algorithm for diffractive imaging. *Ultramicroscopy* **109**, 1256-1262 (2009).
14. Lebeau, J. M., Findlay, S. D., Allen, L. J. & Stemmer, S. Quantitative atomic resolution scanning transmission electron microscopy. *Phys. Rev. Letts.* **100**, 206101 (2008).
